# Extraction and Encapsulation of Phytocompounds of Poniol Fruit via Co-Crystallization: Physicochemical Properties and Characterization

**DOI:** 10.3390/molecules28124764

**Published:** 2023-06-14

**Authors:** N. Afzal Ali, Kshirod Kumar Dash, Vinay Kumar Pandey, Anjali Tripathi, Shaikh Ayaz Mukarram, Endre Harsányi, Béla Kovács

**Affiliations:** 1 School of Agro and Rural Technology, IIT Guwahati, Guwahati 781039, Assam, India; 2 Department of Food Processing Technology, Ghani Khan Choudhury Institute of Engineering and Technology (GKCIET), Malda 732141, West Bengal, India; 3 Department of Bioengineering, Integral University, Lucknow 226026, Uttar Pradesh, India; 4 Department of Biotechnology, Axis Institute of Higher Education, Kanpur 208001, Uttar Pradesh, India; 5 Faculty of Agriculture, Food Science and Environmental Management, Institute of Food Science, University of Debrecen, 4032 Debrecen, Hungary; ayaz.shaikh@agr.unideb.hu; 6 Faculty of Agriculture, Food Science and Environmental Management, Institute of Land Utilization, Engineering and Precision Technology, University of Debrecen, 4032 Debrecen, Hungary

**Keywords:** Poniol extract, phytocompounds, sucrose, co-crystallization, encapsulation

## Abstract

Poniol (*Flacourtia jangomas*) has beneficial health effects due to its high polyphenolic and good antioxidant activity content. This study aimed to encapsulate the Poniol fruit ethanolic extract to the sucrose matrix using the co-crystallization process and analyze the physicochemical properties of the co-crystalized product. The physicochemical property characterization of the sucrose co-crystallized with the Poniol extract (CC-PE) and the recrystallized sucrose (RC) samples was carried out through analyzing the total phenolic content (TPC), antioxidant activity, loading capacity, entrapment yield, bulk and traped densities, hygroscopicity, solubilization time, flowability, DSC, XRD, FTIR, and SEM. The result revealed that the CC-PE product had a good entrapment yield (76.38%) and could retain the TPC (29.25 mg GAE/100 g) and antioxidant properties (65.10%) even after the co-crystallization process. Compared to the RC sample, the results also showed that the CC-PE had relatively higher flowability and bulk density, lower hygroscopicity, and solubilization time, which are desirable properties for a powder product. The SEM analysis showed that the CC-PE sample has cavities or pores in the sucrose cubic crystals, which proposed that the entrapment was better. The XRD, DSC, and FTIR analyses also showed no changes in the sucrose crystal structure, thermal properties, and functional group bonding structure, respectively. From the results, we can conclude that co-crystallization increased sucrose’s functional properties, and the co-crystallized product can be used as a carrier for phytochemical compounds. The CC-PE product with improved properties can also be utilized to develop nutraceuticals, functional foods, and pharmaceuticals.

## 1. Introduction

Poniol (local Assamese dialect) (*Flacourtia jangomas* Lour.) belongs to the family Salicaceae and is a fruit-bearing tropical plant. It is an underutilized plant distributed in the Brahmaputra valley and northeast part of India; it probably migrated from upper Myanmar and Bangladesh and has been cultivated as a rare plant [1]. The fruits are palatable, bright in color, and eaten raw during the summer season when they are ripened. The fruit contains magnesium, potassium, calcium, manganese, and aluminum and is high in sodium (139.32 mg/100 g) [2]. The fruit is used to treat bilious conditions and diarrhea and has antidiabetic and cytotoxic properties [3]. According to existing research, the Poniol plant’s fruits have astringent, anti-inflammatory, diaphoretic, and antibacterial characteristics, and they are used to treat illnesses such as asthma, diarrhea, jaundice, liver-related diseases, nausea, biliousness, and diabetes [4,5].

Co-crystallization is a new encapsulation process utilizing sucrose as a matrix for incorporating bioactive food components. Co-crystallization is a good alternative as it is flexible and economical for incorporating active compounds into powdered foods. The crystalline structure of sucrose gets modified from perfect shape to irregular agglomerate crystals to form a porous matrix where the target active ingredient is incorporated. The agglomerate appears sponge-like, having void space and a high surface area [6]. Supersaturated sucrose syrup spontaneously crystallizes at elevated temperatures (>120 °C) and low moisture (3–5%, or dissolved solids of 95–97 Brix) [7]. If a second ingredient is added simultaneously, the second ingredient is incorporated into the void spaces within the agglomerates of micro-sized crystals with a size smaller than 30 mm via random crystallization. Co-crystallization enhances the solubility, wettability, homogeneity, dispersibility, hydration, anticaking, stability, and flowability of the encapsulated bioactive compound. It also has advantages such as the ability to transform liquid form (core material) to powdered form without drying, and the agglomerate structure ensures direct tableting characteristics of the substance, which is beneficial to the candy and pharmaceutical industries [8]. The encapsulation of plant components in sucrose through the co-crystallization process has been reported for pomegranate peel extract [9], aqueous ethanol extract of unused Chokeberries [10], carotenoids [11], catechin or curcumin [12], soluble fiber [13], catechin or curcumin pre-emulsified with soybean protein isolate [12], ginger oleoresin [14], and *Securigera securidaca* seed extract [15].

The encapsulation of Jamaica (*Hibiscus Sabdarifa* L) granules through co-crystallization improved the developed product’s solubility, dispersibility, and homogeneity. Co-crystallization process increased the entrapment yield and preserved the antioxidant activity of yerba mate (*Ilex paraguariensis*) extract [16]. The co-crystallization of natural antioxidant yerba mate extract with sucrose for the development of compressed tablets showed fast release in an aqueous medium, constituting a useful method for the oral delivery of active compounds with health benefits [17]. Few researchers developed solid dosage forms containing zinc (17 mg/g) obtained through co-crystallization in a sucrose matrix to increase zinc nutrition in high-risk populations. The resulting product had a high encapsulation efficiency (98%) and was stable. Encapsulation of yerba mate extract containing caffeoyl derivatives and flavonoids through co-crystallizing in a supersaturated sucrose solution had a standard cluster-like agglomerate structure with void spaces and sucrose crystal sizes ranging from 2 to 30 mm [18]. Co-crystallization of calcium lactate, magnesium sulfate, and yerba mate extracts in a supersaturated sucrose solution reduced the hygroscopic characteristics of yerba mate extracts without affecting their high solubility [9]. The study showed that the sucrose matrix mainly influenced the flowability, solubility, density, and size distribution of co-crystallized products. The water activity and hygroscopicity depended on the active ingredient added to the co-crystallized product.

Although there are limited scientific reports on the Poniol plant, several studies revealed the fruit extract presented several health benefits. The extracts of the different Poniol plant parts demonstrated cytotoxic, analgesic, antidiabetic, antidiarrheal, antimicrobial, and analgesic properties [1]. Due to the presence of ascorbic acid (24.00 mg 100 g^−1^) and phenol (1.28 mg 100 g^−1^), the fruit displayed a considerable quantity of antioxidant activity (8.93% mg^−1^) [19]. The vitamin C (15.21–223.25 mg 100 g^−1^), soluble sugar (13.77%), and vitamin B complex of riboflavin (236.84 µg 100 g^−1^) and thiamine (42.97 µg 100 g^−1^)-reducing sugar (2.15–9.82%) present in the fruit has also been reported [19,20,21]. The plant fruit’s aqueous, methanol, and ethanol extracts showed strong antimicrobial activity against *Klebsiella pneumonia*, *Escherichia coli*, and *Pseudomonas aeruginosa* [22]. Compared to the standard drug, chloramphenicol, the n-butanol extract from the plant fruits had strong antibacterial efficacy against *S. aureus* and *Escherichia coli* [23]. Saikia et al. (2016) observed that the acetone extract of fresh fruits exhibited a total phenolic content of 377.00 mg (mg GAE/100 g) and a total flavonoid content of 6.66 mg (mg QE/100 g). Their study also revealed a metal chelation capability of 18.55% and DPPH radical scavenging activity above 90% [24]. So, developing functional foods via incorporating this underutilized plant fruit extract could be a beneficial approach for valorizing this plant and its health benefits. With the above facts, the objective of this study was to encapsulate the Poniol fruit ethanolic extract using the co-crystallization process and analyze the physicochemical properties of the co-crystalized product.

## 2. Results and Discussion

### 2.1. Antioxidant Activity, Entrapment Yield, and Loading Capacity of the Co-Crystallized (Cc-Pe) Sample

The initial phenolic content (L_0_) in the crude PE was 38.17 (mg GAE/100 g). The antioxidant activity (DPPH) of co-crystallized powder (Table 1) was 65.10%, and there was a 30.32% reduction in antioxidant activity compared to the crude PE (98.42%). Exposure to heat during the co-crystallization process (60 °C) may lead to the degradation of some polyphenols as well as a lower TPC in the CC-PE powder compared to the PE.

The loading capacity (L_c_) is defined as the amount of TPC loaded in one gram of co-crystallized material and was found to be around 29.25 (mg GAE/100 g) for the CC-PE sample (Table 1). The observed lower Lc and the DPPH values show a strong relationship between the phenolic content and antioxidant activity. The entrapment yield of the sucrose CC-PE was 76.38%. Sardar and Singhal (2013) reported an entrapment yield of 35.23% (for 1,8-cineole) and 67.18% (for α-terpinyl acetate) for encapsulating cardamom oleoresin in sucrose [4]. The entrapment yield (%) in our study was found to be reasonably higher due to the fact that the higher $L_{C}$ resulted from the greater retainment of the TPC and the higher crude extract (PE) quantity (in mL) incorporated during the co-crystallization process. Behnamnik et al. (2019) reported an antioxidant capacity of 56.74% for the product of *Securigera securidaca* (L.) seed extract co-crystallized with sucrose [25], which is lower than our observed antioxidant value. Therefore, the comparatively better entrapment yield and the retainment of antioxidant activity after the co-crystallization process suggest that the CC-PE can be used as a functional food since it carries polyphenolic compounds that promote health. Polyphenolics are very important in promoting health and, at the same time, treating diseases [26,27,28,29].

### 2.2. Bulk Density and Tapped Density

The bulk volume was measured after manually tapping the cylinder two times on a flat tabletop surface. The powder poured into a cylinder will have a particular bulk density. The tapped density denotes the powder’s bulk density after a specific compaction process, generally involving the vibration of the container. The bulk density (Table 1) of the CC-PE (0.723 g/cm^3^) was found to be slightly higher than dried RC powder, whose value was 0.716 (g/cm^3^). The filling up of the interstices/crevices by the PE (where the total soluble solid content is 2%) might increase the solid concentration of the CC-PE agglomerate powder, decreasing the bulk volume and increasing the bulk density. No significant differences (*p* > 0.05) were observed for the bulk densities between the RC and CC-PE powder samples. Bulk density indicates the material’s quantity required to fill a specific package volume, hence providing valuable information regarding handling and logistic applications [30]. Thus, the observed values of the bulk densities of the CC-PE powder confirmed a better packaging performance than the RC powder due to the lesser space requirement for packaging. The parameters that affect it are primarily the material components of the powder product, the humidity and temperature conditions of the environment, and the particle size [31]. The tapped density of the CC-PE (Table 2) powder (0.748 g/cm^3^) was slightly lower than the RC powder (0.774 g/cm^3^). The more crystalline nature of the PS, which was confirmed via the XRD analysis, could provide more compactness of the RC molecules. Additionally, the regular sugar crystal sizes could provide better alignment/rearrangements of sucrose crystals when tapping and occupying limited spaces between the sucrose powder particles. The more irregular shapes of the CC-PE crystals, which were confirmed via the formation of sucrose crystal agglomerates, made the rearrangement/alignment of the sucrose crystals more difficult and occupied more space between the sucrose powder particles. The SEM micrographs also confirmed the CC-PE powder’s irregular crystal size/morphology. The irregular morphology of the CC-PE powder mass might possibly hold more spaces, which would, in turn, occupy relatively more volume than an equivalent RC powder mass. Since the tapped volume is inversely proportional to the tapped density, occupying more volume might reduce the tapped density of the CC-PE powder compared to the RC powder. No significant differences (*p* > 0.05) were observed for the tapped densities between the RC and CC-PE powder samples. A decrease in the tapped density, whose values ranged from 0.77–1.00 (g/cm^3^), has been reported for the propolis extract co-crystallized with sucrose when the propolis ethanolic extract amount increased from 10 to 40 mL [32] and soluble fiber co-crystallized with sucrose (0.57 g/cm^3^) [13]. The bulk and the tapped density values were significant for the determination of the flowability property of the powder samples.

### 2.3. Flowability

The ability of a powder to flow is called powder flowability. Flowability is the result of the combination of material physical properties that affect the material flow. The powder material handling and the caking properties during storage mainly depend on the powder flowability characteristics. The flowability was determined using Hausner’s ratio (HR) for the dried co-crystallized powder. Hausner’s ratio measures inter-particulate friction and is defined as the ratio of tapped density to the bulk density of the co-crystallized powder. HR < 1.11 is considered excellent flow, while HR > 1.60 is regarded as extremely poor flow; in terms of intermediate values, HR between 1.12–1.18 is considered good flow, HR between 1.19–1.25 is considered passable flow, HR between 1.35–1.45 is considered poor flow, and HR between 1.46–1.54 is considered very poor flow [33,34]. The flowability of the co-crystallized powder (CC-PE) of HR 1.082 was better than the recrystallized sucrose (RC) crystal, which has an HR value of 1.034 (Table 2). The RC sucrose powder hygroscopicity was also slightly higher as compared to the co-crystallized powder. A slight increase in the hygroscopicity of the RC powder would decrease the powder’s flowability by increasing the powder’s stickiness, resulting in the formation of large sugar agglomerates. This indicates that the co-crystallization process improved the flowability property of the co-crystallized (sucrose + Poniol extract) powder. The improved flowability property (Hausner ratio values ~1) of co-crystallized sucrose with yerba mate extract has been reported [16].

### 2.4. Hygroscopicity

The tendency of powder to attract and hold water molecules from the surrounding environment is term as hygroscopicity. Hygroscopicity was determined as the weight gained by the products after seven days of their formation reached equilibrium when stored under the environmental conditions of RH = 75% and at 25 ℃. This property is an essential factor for defining the product’s packaging prerequisites. The hygroscopicity of the dried CC-PE powder (Table 2) was 11.67%. It showed a significant (*p* < 0.05) decrease in the hygroscopicity compared to the recrystallized sucrose (RC), which has a value of 12.34%. The moisture content of any product determines the degree of spoilage or shelf of any product since any biochemical reaction is mainly dependent on the moisture content. The decreased hygroscopicity of the co-crystallized powder indicated that the product would absorb less moisture during the storage period than the RC sucrose powder, thereby prolonging the product’s shelf life. Higher hygroscopicity negatively influences the powder’s flowability by increasing the powder’s stickiness, forming large sugar agglomerates. The sugar agglomerates’ chain reaction forms thick masses of sugar powder, which is a very undesirable property of any powder material. Similar findings of the lower hygroscopicity of the sucrose matrix have also been reported when co-crystallized with carotenoids extracted from carrots and found in yerba mate extract [11].

### 2.5. Solubilization Time

Solubilization time is the time of dissolving the solute (co-crystallized powder) in a solvent (water) to form a homogenous solution (Table 2). The solubilization time of RC powder was found to be around 64.8 s, which signifies that the Brix of the solution did not change (9 Brix), indicating the crystal powder was fully dissolved in the solvent (water). The solubilization time of the CC-PE powder in water was found to be 62.5 s, which was lower than the RC powder. This shows that the co-crystallization process slightly decreased the solubilization time of the product. No significant differences (*p* > 0.05) were observed between the RC and the CC-PE powder samples. Greater amorphousness with reduced crystallinity, confirmed via the XRD analysis, might trigger the reduced solubilization time of the CC-PE. The higher crystalline RC would increase the compactness of the sucrose molecules. Obviously, the more amorphous material could possess more hydration power. This probably could enable quick solvent (distilled water) migration through the agglomerate pores of CC-PE, permitting the active compounds (e.g., PE) residing in the intervening spaces between crystals to release rapidly, thereby increasing solubilization power. A decrease in the solubilization time of sucrose co-crystallized with yerba mate extract (1.33 min) when compared to RC sucrose (1.37 min) has also been reported [31]. A similar report on the decrease in the solubilization time of sucrose matrix co-crystallized with yerba mate extract has also been revealed [16]. It can be concluded that there was a slight tendency for better solubility for the CC-PE, which is beneficial when serving the product.

### 2.6. X-ray Diffraction Analysis

**Figure 1:**
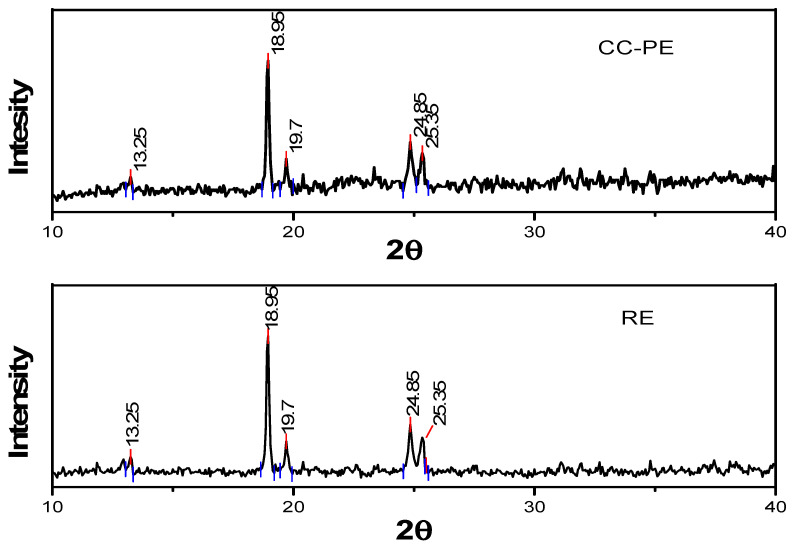
X-ray diffractograms of the RC (recrystallized sucrose) and CC-PE (sucrose co-crystallized with Poniol extract).

The XRD diffractograms of the RC sugar and the CC-PE powder are shown in Figure 1. Both the samples showed significant peaks at the 2θ values of 13.2, 18.9, 19.7, 24.8, and 25.3. The graph of the co-crystallized powder did not show any shift in the 2θ values from the RC sucrose values. However, the intensities of the peaks of the RC sucrose were higher than those of the co-crystallized powder sample. The co-crystallized powder did not show any new distinct peaks with higher intensities in the diffraction graph, which confirmed that the extract was not crystallized after the co-crystallization process and was incorporated effectively in the voids of the sucrose. Additionally, the non-crystalline nature of the PE might not influence the crystal structure of the sugar during the co-crystallization process. The degree of crystallinity of the CC-PE was 37.8%, while the RC powder had 51.9% crystallinity. This higher crystallinity of RC sugar was also supported by the DSC analysis that showed that the RC sucrose had a higher endothermic melting peak with a higher intensity as compared to the co-crystallized powder. The XRD diffraction pattern verified that the co-crystallization process reduced the degree of crystallinity of the sucrose, which could be due to the amorphous nature of the extract. Any material had been classified as high crystalline when above 50%, medium crystalline if 20–50%, and low if 20% [35]. Accordingly, the XRD diffractograms proved that the RC sucrose was a crystalline compound while the co-crystalline powder showed medium crystalline properties. The XRD analysis concluded that the co-crystallization treatment did not change the overall crystal structure of the sucrose, although the crystallinity had been reduced. Reports on the non-occurrence of the additional peaks with higher intensities were reported similarly for co-crystallized sucrose with *Basella rubra* extract, yerba mate extract, and cardamom oleoresin [6,16,36]. The decrease in the crystallinity of the co-crystallized product as compared to sucrose has also been reported for vitamin-B12-fortified co-crystallized sugar cubes [37].

### 2.7. Microstructure of Microencapsulated PONIOL (F. Jangomas) Powder after Co-Crystallization

**Figure 2:**
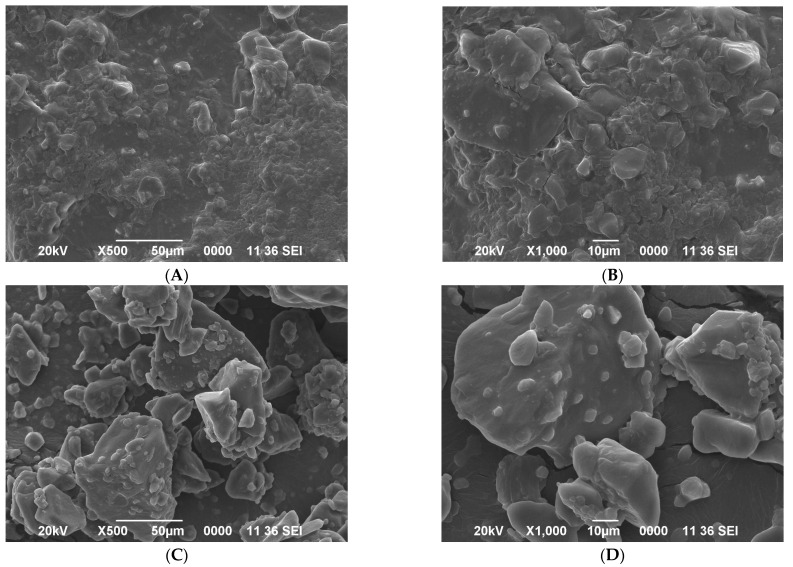
SEM microphotographs of the RC ((**A**) = 500× and (**B**) = 1000× magnification) and the CC-PE ((**C**) = 500× and (**D**) = 1000× magnification).

The microcapsules’ surface morphology and internal structure were investigated by analyzing the scanning electron microscope (SEM) images (Figure 2). The RC sucrose (Figure 2A,B) showed a relatively regular crystal shape, which could be attributed to the proper alignment of atoms in the RC sucrose molecule. In contrast, the CC-PE powder (Figure 2C,D) exhibited an irregular agglomerated crystal, giving a porous structure that could possibly produce bigger cohesive solids than the original fine RC powder granules.

The porous configuration of the sucrose potentially produced through the co-crystallization process could accept a second ingredient (extract), enabling the incorporation of the second active ingredient [38]. The addition of the Poniol extract during the spontaneous crystallization of the supersaturated sucrose/sugar syrup that had occurred when heated (above 120 °C; 95–97 Brix) resulted in the incorporation of the second ingredient into the void spaces/irregular cavities of the micro-sized crystal agglomerates [35]. The presence of agglomerated clusters in the co-crystallized powder indicated the better entrapment/incorporation of Poniol extract into the recrystallized sucrose. Reports on better entrapment due to the presence of the agglomerated sucrose clusters have also been reported for sucrose crystals that were co-crystallized with yerba mate extract, Basella rubra extract, and carotenoids extracted from carrot [6,11,16]. The SEM analysis provided important pieces of information about the surface morphological characteristics, which are very important for both the products’ hygroscopicity and flowability properties.

### 2.8. Fourier Transform Infrared (FT-IR) Spectroscopy

**Figure 3:**
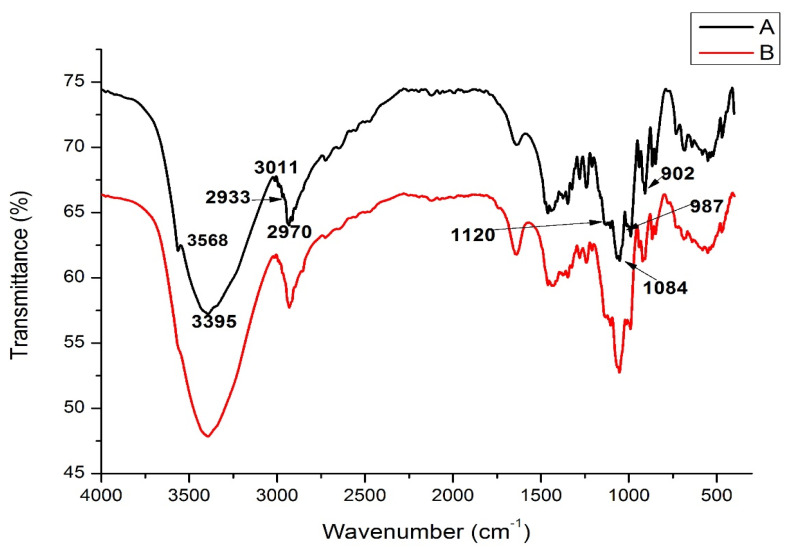
FT-IR spectra of (A) RC and (B) CC-PE.

The FT-IR analysis analyzed the chemical structure and the functional groups of the RC sucrose and the co-crystallized powder with Poniol extract in the range of 400–4000 cm^−1^ wavenumbers (Figure 3). The significant peaks found at 3568 cm^−1^ and 3395 cm^−1^ were assigned to the -OH groups’ vibration (stretching). Bands attributed to the stretching of C-H groups were detected around 3011 cm^−1^ and 2970 cm^−1^. The characteristic peak for -CH_2_ was found at around 2933 cm^−1^, and for C-O groups, it was found at around 1120 cm^−1^ and 987 cm^−1,^ respectively. The C-O stretching vibration modes were found at around 1084 cm^−1^ and 902 cm^−1^. The band between 800–1500 ${\mathrm{cm}}^{-1}\;$ has been called the ‘fingerprint’ for sucrose (sugars), emergence from the CO-stretching vibrations, and the CH_2_ groups’ symmetrical deformation, thereby offering high structural information [39,40,41,42,43,44].

A similar chemical bonding structure with respect to the wavenumber, as mentioned above, for sucrose has been reported [18,30,32]. The co-crystallized powder had no significant additional peaks compared to the RC sucrose. This means there were no additional conformational changes in the chemical bonding structure during the co-crystallization process. However, there was an increase in the peak intensities at the band of $\sim 1650{\;\mathrm{cm}}^{-1}$, a wavenumber assigned to the -OH bending vibration of water molecules. It was possible to overlap this transmittance peak ($\sim 1640{\;\mathrm{cm}}^{-1}$) with antisymmetric (COO−) stretching bands [42]. Therefore, it can be suggested that the overlapping of -OH bending vibration and the stretching vibrations of C=C and C=O of polyphenolic compounds (from the extract) might increase the intensity of the $\sim 1650{\;\mathrm{cm}}^{-1}$ band [32,45]. From the FTIR analysis, it can be suggested that there was a good incorporation/entrapment of the extract to the sucrose matrix since no significant new peaks were observed compared to the RC powder sample.

### 2.9. Thermal (DSC) Analysis of Co-Crystallized Poniol Extract Powder

**Figure 4:**
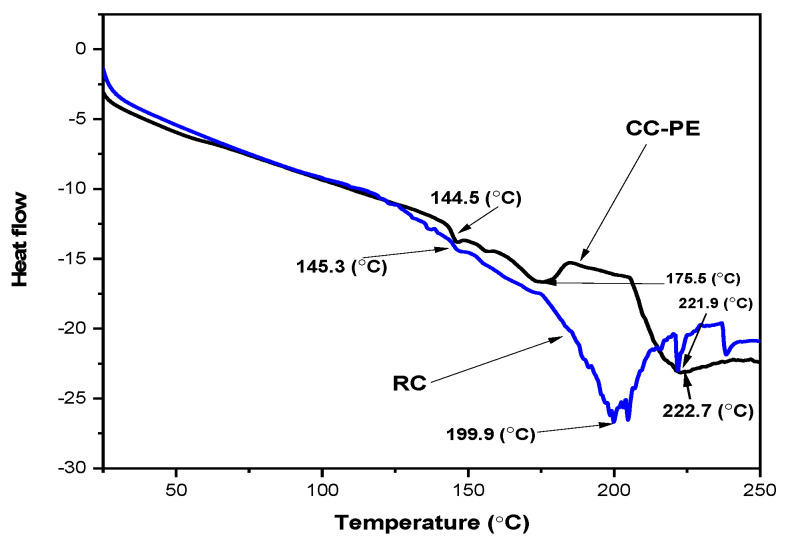
DSC thermograms of the RC and CC-PE samples.

Differential scanning calorimetry (DSC) measures the temperatures and heat flows associated with transitions in materials as a function of time and temperature in a controlled atmosphere. These measurements offer information about physical and chemical changes involving endothermic or exothermic processes or changes in heat capacity. Glass transition temperature (Tg) is a characteristic parameter of a material above which it behaves like a liquid (rubbery state). There was a shift in the Tg value of the co-crystallized powder (144.5 °C) from the RC sucrose (145.3 °C). The DSC thermograms for the RC sucrose and the co-crystalized samples are depicted in Figure 4.

The RC sucrose thermogram showed two significant endothermic peaks at approximately 199.9 °C and 221.9 °C, while the co-crystallized powder showed a distinct peak at 175.5 °C. The first peak (199.9 °C) of the RC sucrose was due to the melting point of the sucrose, while the second peak could be assigned to sucrose degradation [31]. The first peak of the co-crystallized powder might be due to the fusion of the sucrose and the extract [46]. Figure 4 shows that the endothermic peaks were shifting. The peaks of the co-crystallized powder sample (both 175 °C and 222.7 °C) were broad with a lower intensity, signifying the effect of the extract entrapped in the powder. The lower crystallinity explained via the XRD property analysis of the co-crystallized power supported this effect. The shifting and total or partial disappearance of the co-crystallized powder’s thermal events (melting point) could be taken as proof of its good incorporation into the matrix, indicating good incorporation of the extract into the sucrose matrix. The second endothermic transition at 222.7 °C could be attributed to the degradation of all the components in the CC-PE powder [16,47,48,49]. The retainment of the crystalline state, as evidenced by the melting endotherms of the DSC analysis, has also been reported in the sucrose co-crystallized with yerba mate extract [50].

## 3. Material and Methods

### 3.1. Raw Material and Chemicals and Reagents

Fresh Poniol (*Flacourtia jangomas*) fruit was obtained from the local market of Udalguri district, Assam, India, and stored at 4 °C for further use. Distilled water was used for washing of the fruit. All the chemicals and reagents were of analytical grade. The ethanol (99.9%) was supplied by Changshu Hongsheng Fine Chemicals Co., Ltd. (Changshu, China). Folin & Ciocalteu’s phenol reagent (2.0 N) and gallic acid (98%) were supplied by Sisco Research Laboratories Pvt. Ltd. (Taloja, Maharashtra, India). Sucrose (AR grade), sodium carbonate anhydrous (99.50–100.50%), and 2,2-Diphenyl-1-picrylhydrazyl or DPPH (85.0%) were supplied by Himedia Laboratories Pvt. Ltd. (LBS Marg, Mumbai, India). Ascorbic acid (99.0%) was purchased from Merck Life Science Pvt. Ltd. (Vikroli East, Mumbai, India). Deionized water was used to prepare all the chemical solutions.

### 3.2. Fruit Extract Preparation

The whole fruit was dried in a domestic microwave oven (IFB, 20BC4, India) at 720 W power, followed by grinding. The dried powdered sample (100 g) was put in a conical flask and dissolved in ethanol (900 mL, 60%) for extraction closed with cotton. The extraction was performed through keeping the solution mixture (ethanol + sample) in a shaker (120 rpm) incubator (Remi RIS 24 plus, India) for 48 h at 37 °C [51,52]. After 48 h, the solution was removed, and filtration was carried out through a cheesecloth to collect the filtrate. The filtrate was again centrifuged (8000 rpm for 10 min), and the supernatant Poniol extract (PE) was collected. The extract was stored at refrigerated conditions (−20 °C) for further applications.

### 3.3. Preparation of the Co-Crysta–Llization Products

The sucrose (50 g) solution (70 Brix in distilled water) was heated in a glass beaker at 127 ± 4 °C on a hot plate under intermittent manual mixing until the solution reached 95.8 Brix. The obtained solution was cooled, and when it reached 60 °C, the previously prepared PE or pure ethanol (30 mL) was added, followed by covering it with aluminum foil [9,53]. The obtained supersaturated solution was cooled through dipping the beaker in ice-cold water. This cooling was done in order to prevent long-term exposure of the Poniol extract to high temperatures, since this is known to provoke a browning reaction and flavor degradation. Secondly, the product was to increase the supersaturation of the sugars, which facilitates quicker crystallization. The concentrated sucrose syrup was kept for 72 h at room temperature for the formation of crystals. After that, the sucrose co-crystallized with the Poniol extract (CC-PE) was collected and dried in a hot air oven (Universal, JSGW-TC344) at 40 °C for 15 h and then was ground and sieved through a 500 mm mesh. Blends of raw sucrose, distilled water, and ethanol were crystallized as described above for control. This recrystallized sucrose (RC) was referred to as the control sample.

The resultant powder’s material characterization was carried out through analyzing phenolic content, scavenging activity, color difference, solubility, hygroscopicity, loading capacity and entrapment yield and flowability, DSC, XRD, and SEM for the CC-PE and the control samples.

#### 3.3.1. Determination of Total Phenolic Content

The total phenolic content (TPC) analysis was conducted according to the Folin–Ciocalteu assay [54] to evaluate and study the influence of the co-crystallization process in the TPC content among the PE, CC-PE, and control samples. The CC-PE powder (5 mg) was dissolved in 5 mL of ethanol (60% *v*/*v*) and vortexed, followed by keeping it at room temperature (25 °C) for 30 min. The concentration of the standard solution of gallic acid ranged from 0.01–0.5 mg/mL. 200 µL of each pure ethanol PE and the solutions of CC-PE and the control powder were pipetted out in different test tubes, followed by adding distilled water (800 µL) and 5 mL Folin–Ciocalteu (10%). The solution mixture was vortexed and kept for 1 min, and then 4 mL sodium carbonate (7.5%) was added. The solution was incubated for 30 min at room temperature in dark conditions. The absorbance was measured at 765 nm wavelength in a UV spectrophotometer (Cary 100, Agilent Technologies, Santa Clara, CA, USA), and the phenolic content was expressed in mg GAE/100 g of dried fruit.

#### 3.3.2. Determination of Antioxidant Activity

Free radical scavenging activity towards the DPPH reagent method was used to determine the antioxidant activity of the samples [55,56]. Co-crystallized powder (0.5 g) was dissolved in 5 mL of ethanol (60% *v*/*v*), followed by vortexing, and was kept at room temperature (25 °C) for 30 min. The RC powder was dissolved via the same procedure as the CC-PE for the control sample preparation. 100µL of each extract/solution and the pure ethanol (60%) were mixed with 3.9 mL of ethanolic solution of DPPH (25 mg/L). Then, the mixture was incubated for 30 min in the dark, and absorbance was measured at 517 nm using a UV-VIS spectrophotometer (Cary 100, Agilent Technologies, Santa Clara, CA, USA). The percentage of inhibition (*I %*) of the DPPH free radical expressed the antioxidant activity and was calculated using the formula:

(1) $$DPPH\;\left(\%\;I\right)=\left(1-\frac{A_{s}}{A_{o}}\right)\times 100$$

where

$A_{s}$ = Absorbance of control (DPPH solution without extract)

$A_{o}$ = Absorbance of sample.

#### 3.3.3. Loading Capacity and Entrapment Yield [11]

Loading capacity (Lc) can be calculated as the total phenolic content of the Poniol extract loaded in 1 g of co-crystallized material expressed as mg GA/g powder.

The entrapment yield (*EY*) was calculated as follows:

(2) $$EY\;\left(\%\right)=\left(\frac{L_{c}}{L_{0}}\right)\times 100$$

where $L_{0}$ was the initial TPC of the Poniol extract expressed as mg GA/g dried Poniol fruit.

#### 3.3.4. Bulk Density, Tapped Density, and Flowability

The bulk density (g/cm^3^) was calculated through measuring the volume occupied by the known masses of the RC and the RC-PE powder samples that were filled under gravity in a graduated cylinder without compacting. For estimation of tapped density (g/cm^3^), the volume measurements were made following 10, 25, and 50 manual taps. Each sample was replicated in triplicate, and the obtained values were averaged.

The flowability of the co-crystallized powders was determined using Hausner’s ratio (HR). The value of HR was calculated according to the ratio of the tapped density to the bulk density [3].

The Hausner’s ratio was calculated as:

(3) $$HR=\frac{\rho_{T}}{\rho_{B}}$$

where $\rho_{T}$ was the tapped density and $\rho_{B}$ was the bulk density of the product.

#### 3.3.5. Hygroscopicity

The tendency of powder to attract and hold water molecules from the surrounding environment is termed hygroscopicity. The hygroscopicity of both samples was determined through adopting methods described by [57,58] with slight modifications. Samples (1 g) were kept inside the desiccator, which contains sodium chloride solution (RH 75.3%). The result was calculated as the mass of water absorbed per 100 g of the sample after seven days of storage.

(4) $$Hygroscopicity=\frac{Weight\;of\;moisture\;absorbed\left(g\right)}{Initial\;weight\;of\;the\;sample\left(g\right)}$$

#### 3.3.6. Solubilization Time

The dried sample (both RC and CC-PE) powder (1 g) was blended with distilled water (10 mL) at 25 °C with continuous stirring [32]. Aliquots of the sugar solutions were taken in a refractometer at regular intervals and the Brix changes were observed. The solubilization time was determined through observing the time (in seconds) when the Brix changes became static.

#### 3.3.7. X-ray Diffraction (XRD)

An X-ray diffractometer (STOE, Darmstadt, Germany) equipped with a Miniflex goniometer in reflection X-ray source: copper anode (Cu Kα = 1.5418 Å), current = 15 mA (fixed), voltage = 30 kV (fixed), provided via a copper Kβ filter was used to analyze the crystallinity of the samples. The samples were scanned between the 2θ values from 10° to 40° [6].

#### 3.3.8. Scanning Electron Microscopy (SEM)

Scanning electron microscopy (JEOLJSM-6390LV, Japan) was used to analyze the microstructure of the RC and CC-PE powder samples. The co-crystallized powder was attached to the SEM stubs using two-sided adhesives tape and then coated with a layer of gold (40–50 nm) and analyzed using a 20 kV acceleration voltage.

#### 3.3.9. FTIR (Fourier Transform Infrared Spectroscopy) Analysis

The chemical bonding and functional groups of the RC and CC-PE powders were analyzed using Fourier transform infrared spectroscopy (Impact −410, Nicolet, Alexandria, USA) and the spectral analysis was performed with the software OMNIC E.S.P.5.0. in the wavenumber range of (400–4000) cm^−1^ at a resolution of 4 cm^−1^ [25,32].

#### 3.3.10. Thermal Analysis (Differential Scanning Calorimetry)

Differential scanning calorimetry (DSC-60, SHIMADZU) with TA-60 WS software was used for the DSC analysis of the RC and CC-PE powder samples. Samples (4–6) mg were placed in aluminum pans and then hermetically sealed. An empty pan was used as the reference. The temperature range for the samples to be heated was (25 to 250) °C and the heating rate was 10 °C/min [30].

### 3.4. Statistical Analysis

The analyses were conducted in triplicate, and the data are presented as mean ± standard deviation. Each parameter underwent an analysis of variance (ANOVA) with Tukey’s test to determine the significance of the effects and interactions between them. A *p* < 0.05 was considered to be statistically significant. Origin software was used to draw the graphs.

## 4. Conclusions

The ethanolic extract of Poniol (*Flacourtia jangomas*) fruit was encapsulated using a co-crystallization process with sucrose, and the physicochemical properties of the co-crystalized product were analyzed in this study. The obtained CC-PE and RC products’ material characterization was carried out through analyzing the TPC, antioxidant activity, loading capacity, entrapment yield, bulk and tapped densities, hygroscopicity, solubilization time, flowability, DSC, XRD, FTIR, and SEM. The bulk density and the flowability of the CC-PE sample were 0.723 and 1.082 HR, respectively, which were higher than the RC sample, whose values were 0.716 and 1.034 HR, respectively. The bulk hygroscopicity and solubilization time of the CC-PE sample were 11.672% and 62.5 s, respectively, lower than the RC sample, whose values were 12.345% and 64.8 s, respectively. The result revealed that the CC-PE product had a good entrapment yield (76.38%) and could retain the TPC (29.25 mg GAE/100 g) and antioxidant properties (65.10%) even after the co-crystallization process. The higher flowability bulk density and the lower hygroscopicity and solubilization time of the CC-PE sample compared to the RC sample are desirable for a powder product. The XRD, FTIR, and DSC analyses also showed that there were no changes in the crystal structure, functional group bonding structure, and thermal properties of the sucrose, respectively. The SEM analysis showed that the CC-PE sample has cavities or pores in the sucrose cubic crystals, which suggests that the entrapment was better. From the results, we can conclude that co-crystallization increased the sucrose’s functional properties, and the co-crystallized product can be used as a carrier for phytochemical compounds. The obtained CC-PE, which contains bioactive compounds with antioxidant properties, may possibly be used as a natural sweetener with improved properties while considering the verification of consumer acceptance. The CC-PE product with improved properties can also be utilized to develop nutraceuticals, functional foods, and pharmaceuticals.

## Figures and Tables

**Table 1:** Loading capacity, entrapment yield, and antioxidant activity of co-crystallized powder (CC-PE).

Products	Loading Capacity L_C._ (mg GAE/100 g)	TPC (mg GAE/100 g of Dried Fruit)	Entrapment Yield (%)	Antioxidant Activity (%)
CC-PE	29.25 ± 0.03	29.25 ± 0.03	76.38 ± 0.07	65.10 ± 0.03
PE	NA	38.17 ± 0.04	NA	98.42 ± 0.37
Control	0.00 ± 0.00	0.00 ± 0.00	0.00 ± 0.00	1.7 ± 0.03

Values are given as mean ± standard deviation. Similar letters within a column indicate no significant difference (*p* < 0.05). NA = Not applicable.

**Table 2:** Physical properties of the recrystallized sucrose (RC) and the co-crystallized Poniol extract powder.

Sample	Bulk Density	Tapped Density	Flowability (HR)	Hygroscopicity (%)	Solubilization Time (sec)
RC	0.716 ± 0.034	0.774 ± 0.034	1.034 ± 0.042	12.345 ± 0.036	64.8 ± 0.1
CC-PE	0.723 ± 0.023	0.748 ± 0.022	1.082 ± 0.003	11.672 ± 0.023	62.5 ± 0.1

RC = recrystallized sucrose; CC-PE = sucrose co-crystallized with Poniol extract. Values are given as mean ± standard deviation. Similar letters within a column indicate no significant difference (*p* < 0.05).

## Data Availability

The data presented in this study are available on request from the corresponding author.

## Sample Availability

Not Available.
